# Supplementary material for: N-representable one-electron reduced density matrix reconstruction with frozen core electrons
Source: Acta Crystallogr A Found Adv. 2024 Mar 21;80(Pt 3):249–57. doi: 10.1107/S2053273324001645 (PMC11067946; doi:10.1107/S2053273324001645)
Supplement: Supplementary file 1 [file a-80-00249-sup1.pdf]

FOUNDATIONS
ADVANCES

Volume 80 (2024)

Supporting information for article:

N-representable one-electron reduced density matrix reconstruction with frozen core electrons

Sizhuo Yu and Jean-Michel Gillet

SUPPLEMENTARY INFORMATION

A. Fitting score

Here, we list the unweighted fitting scores χ^2 for different data sets and reconstructions. We note $\tilde{\chi}^2_{\text{total}}$ the value of the objective function in the optimization procedure and χ^2_{total} the fitting score computed on the entire range of 0 K, noise-free artificial-data.

Firstly, it can be observed that while the optimal objective function values for the 0 K and 50 K scenarios are similar, the χ^2_{SF} is substantially larger than the $\tilde{\chi}^2_{\text{SF}}$ in the 50 K case, suggesting that the thermal motion is not perfectly accounted for by (**??**). Secondly, in both the 0 K and 50 K cases, restrictions lead to an increase in the optimal fitting score $\tilde{\chi}^2_{\text{total}}$ (for noised 0 K and 50 K data), but a decrease of the χ^2_{total} (noise-free 0 K). Such an observation quantitatively confirms the assumption that restrictions act as regularization, thereby improving reconstruction results.

Scenario	$\tilde{\chi}^2_{\text{SF}}$	$\tilde{\chi}^2_{\text{DCP}}$	$\tilde{\chi}^2_{\text{total}}$	χ^2_{SF}	χ^2_{DCP}	χ^2_{total}
0 K 1% noise	2.683	2.084	4.766	1.798	0.871	2.669
0 K 1% noise sym.	3.090	2.272	5.362	1.336	0.734	2.070
0 K 1% noise sym. & core	3.190	2.271	5.362	1.234	0.741	1.975
50 K 1% noise	2.597	2.258	4.855	22.065	0.896	22.961
50 K 1% noise sym.	2.964	2.662	5.626	17.375	0.724	18.099
50 K 1% noise sym. & core	3.144	2.741	5.885	14.176	0.757	14.933

TABLE S1. $\tilde{\chi}^2_{\text{SF}} = \sum_{\mathbf{q}}(\tilde{F}(\mathbf{q}) - \text{Tr}(\mathbf{PF_q}))^2$ for $|\mathbf{q}| < 0.7$ and $\tilde{F}$ being the 1% noisy artificial-data. Similarly, $\tilde{\chi}^2_{\text{DCP}}$ is the objective function for noisy data. As a reminder, $\tilde{\chi}^2_{\text{total}} = \tilde{\chi}^2_{\text{SF}} + \tilde{\chi}^2_{\text{DCP}}$ is the objective function being optimized, and the noised SF artificial-data is at respectively 0 K and 50 K for two different scenarios. χ^2_{total}, χ^2_{SF}, χ^2_{DCP} refer to the fitting score with respect to the noise-free artificial-data using the entire Ewald sphere up to 1.1 $\mathring{A}^{-1}$(always at 0 K).

B. Mean-field energies

We list the energies evaluated for different reconstructions. Note that the potential energies, hence the total energies, are calculated with a Hartree-Fock Hamiltonian operator, which allows us to calculate an approximate energy with 1-RDMs. For reference, the energies of the DFT 1-RDM with the pob-DZVP basis set are $T_{\text{DFT}} = 223.315$, $E_{\text{DFT}} = -225.005$. However, one should keep in mind that these energies are evaluated with different methods and different basis functions, and are not meant to be compared directly.

Scenario	T	V_{HF}	E_{HF}	Virial ratio
0 K 1% noise	224.554	-447.229	-222.675	0.99581
0 K 1% noise sym.	224.387	-447.546	-223.159	0.99726
0 K 1% noise sym. & core	224.909	-448.237	-223.327	0.99648
50 K 1% noise	223.844	-445.405	-221.561	0.99490
50 K 1% noise sym.	223.676	-446.073	-222.397	0.99714
50 K 1% noise sym. & core	224.884	-447.832	-222.949	0.99570

TABLE S2. The energies and Virial ratios evaluated for different reconstructed 1-RDMs. For potential energies V_{HF}, a mean-field Hartree-Fock energy operator is employed.
